# Editorial: Catalyzing public health leadership research, practice, education, and training

**DOI:** 10.3389/fpubh.2026.1851887

**Published:** 2026-05-04

**Authors:** Howard K. Koh, Barbara X. Wei, Fawn Phelps, Emily M. Burke, William Bean, Louis W. Fry

**Affiliations:** 1 Health Policy and Management, Harvard T.H. Chan School of Public Health, Boston, MA, United States; 2 Association of Schools and Programs of Public Health, Washington, DC, United States; 3 Texas A&M University, Central Texas, Killeen, TX, United States

**Keywords:** mentorship, public health competencies, public health data, public health education, public health leadership, spiritual leadership, values and purpose

## Introduction

The field of public health has always demanded leaders who can act amidst profound uncertainty with commitment, courage and clarity (1). In recent years, those demands have intensified in seemingly unimaginable ways—through the COVID-19 pandemic, rising political polarization, and widening inequities, for example. Public health leaders at every level have faced harassment, burnout, and sustained moral strain while working with limited authority and resources. Assaults on science have eroded trust. Many have been forced to starkly re-examine the fundamental values, purpose and meaning of their work and their lives.

In times like these, public health leadership development must not be left to on-the-job training. Rather, it must be intentionally cultivated, systematically supported, and empirically informed. This Research Topic *Catalyzing public health leadership research, practice, education, and training* advances an integrated approach to future of the field. The 20 articles collectively highlight not only personal and professional concepts for individual leaders but also the mentoring, community partnerships and systems necessary to sustain them.

**Figure 1:**
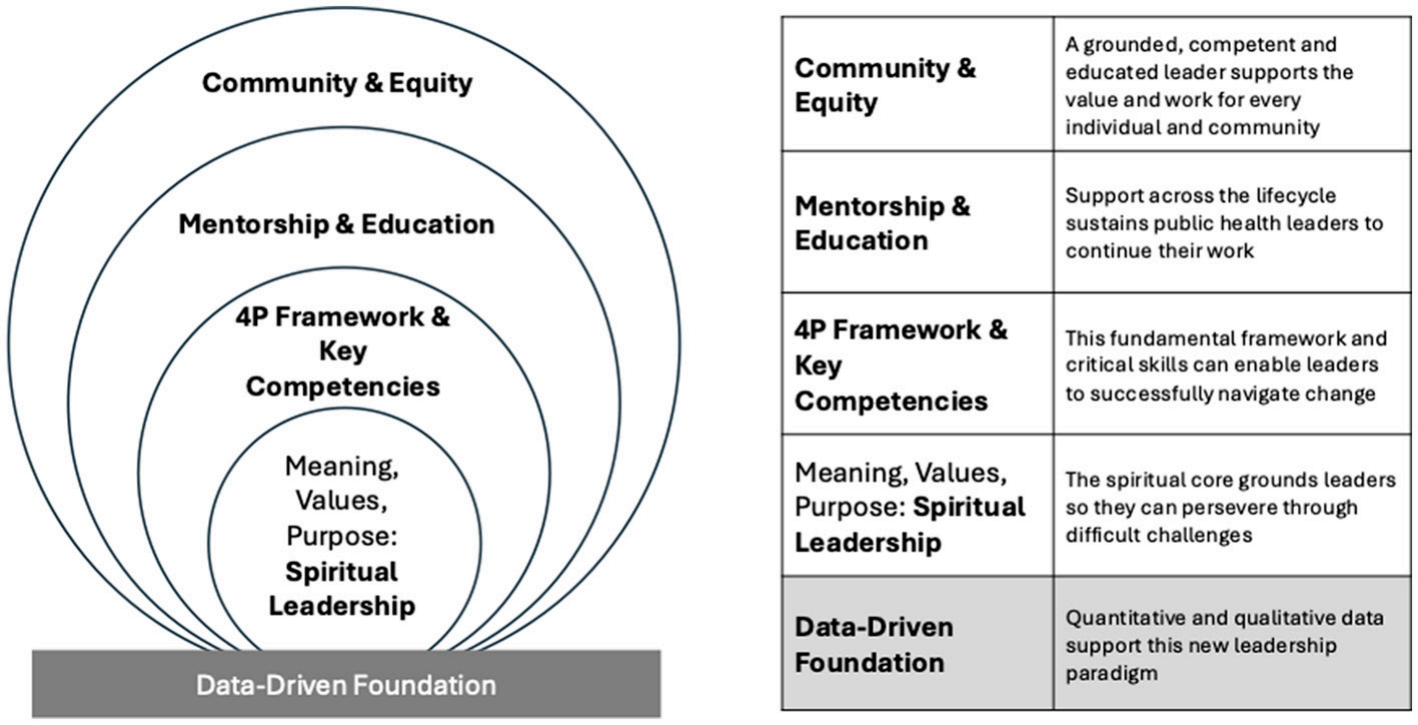
Public health leadership.

Figure 1 offers a way to synthesize these concepts through four nested, mutually reinforcing domains: Meaning, Values and Purpose: Spiritual Leadership; 4P Framework & Key Competencies; Mentorship & Education; and Community & Equity. These domains are not sequential, but rather interdependent and embedded within one another. They describe how public health leadership can be cultivated and sustained through an inner orientation rooted in purpose (Spiritual Leadership); a structural architecture of clearly defined competencies (Key Competencies); intentional formation across the lifespan (Mentorship & Education); and authentic community partnership grounded in equity (Community & Equity). Leadership becomes durable when these dimensions reinforce one another—purpose without competency lacks execution; competencies without community lack legitimacy; mentorship without equity risks exclusion; and community engagement without inner grounding risks fragmentation Empirical studies provide a data-driven foundation to address some of the workforce and organizational challenges.

## Meaning, values, purpose: spiritual leadership

In times of tumult, mindful cultivation of values, purpose, and interconnectedness—for oneself and others—is essential to align personal motivation with collective mission—what Kouzes and Posner refer to as “encouraging the heart” (2). Amidst uncertainty, hope and faith sustain commitment to mission and vision; “hope puts you on the road and faith keeps you there”(3). These themes all contribute to Spiritual Leadership, a concept which has steadily gained traction over recent decades in other leadership research related to organizational management, business and the workplace. In this Research Topic, Fry et al. note how Spiritual Leadership can be relevant to the field of public health in these trying times; it is defined as “the values, attitudes, and behaviors necessary both to motivate and inspire workers and to enhance key individual and organizational goals through a vision of service and a culture based on altruism.” For some, spirituality can involve an organized faith tradition, while for others, it involves other, less formal, ways to experience ultimate meaning, purpose and connection to something bigger then oneself. Gupta has noted that regardless of specific faith worldwide, common emphasis on shared meaning, values and purpose, cut across all cultures (4). Moreover, Fry states that spirituality is required for religion but religion is not required for spirituality. And for many in public health, the field represents a calling (5).

Leadership values of vision, hope/faith, and altruism can foster purpose and meaning through one's work as well as a sense of belonging within a community (Fry et al.). Similar to Fry et al., Chyu et al. emphasize discernment, holistic presence, and reflective self-awareness as foundational to public health leadership, since inner formation shapes outward practice. Purpose informs competency development and strategic direction; belonging is reinforced through mentorship and education; and altruism finds outward expression in community engagement and equity-centered practice. Altruism—expressed through respect, concern and appreciation for both self and others—cultivates trust and relational cohesion. In these ways, Spiritual Leadership can anchor public health in shared vision, mission-centered community, and service.

## Data-driven foundation

Multiple data-driven studies point to the need for more intentional and integrated approaches to public health leadership development. Two contributions analyze data from the Public Health Workforce Interests and Needs Survey (PH WINS), a recurring national survey of state and local public health personnel. Kirkland et al., in analyzing PH WINS alongside the 2022 NACCHO National Profile of Local Health Departments, report that nearly one-third of local health officials are currently new to their roles and tend to be younger and more racially diverse; they place high value on mentorship and organizational support. Hamer et al., also analyzing PH WINS data, identify persistent gaps in data science, data literacy, and data-informed leadership, and emphasize the need to strengthen workforce analytic and strategic competencies. Popalis et al. show that agencies providing formal leadership training and supervisory support have higher measurable employee satisfaction as part of organizational benefit.

Additional empirical work includes the study by Dubois et al., drawing on the National Board of Public Health Examiners' Job Task Analysis of over 2,000 professionals, which finds that leadership is a priority by so many, ranking among the most critical domains in public health practice. Gines et al., in a survey of more than 300 Texas community health workers, identify generational differences in advocacy engagement and mentoring needs, demonstrating the value of supportive leadership in workforce retention. International evidence echoes these findings: Zweigenthal et al. report that South African MPH graduates can exercise leadership, but vary in how such skills are cultivated in formal training. Reng et al. demonstrate gains in leadership capacity through culturally responsive mentorship programs in Nigeria.

Together, these empirical contributions not only document workforce and competency gaps but also the conditions under which public health leadership can be intentionally cultivated through the four nested domains.

## 4 P framework and key competencies

As part of creating a public health leadership national training agenda, Burke et al. offer a comprehensive analysis and synthesis of 79 publications and multiple national standards. They recommend a 4P Framework of Public Health Leadership—Problem, Person, Pathway, and Purpose (Burke et al.)—that positions leadership as multi-level and systems-oriented. Leaders must accurately frame complex public health problems; deepen their personal self-awareness (starting with values and mission); broaden their systems awareness through reflection and developmental coaching; and design pathways for strategic action. By adapting a previously published 3P Framework for Social Innovators (person, problem, pathway) to include the 4th P (purpose), the authors explicitly recognize and acknowledge the spiritual dimensions of public health leadership. In short, leadership involves not just the “what” and the “how” but most importantly the “why” (6). Then as part of national Public Health Leadership Competency Mapping for the training agenda, Burke et al. leverage the 4P Framework to organize 41 leadership competencies into ten thematic areas (Burke et al.). In this way, key competencies of leadership can be operationalized and translated into effective public health action.

Designed to be scalable across academic programs and governmental agencies, the agenda offers a shared language for leadership expectations across career stages. The competencies encompass strategic planning, policy and advocacy, workforce development, communication, data-informed decision-making, resource allocation, equity and social justice, and change management—all essential for contemporary public health practice.

## Mentorship and education

Mentorship and education serve as the developmental bridge for building communities of shared mission to sustain belonging by reinforcing both technical and relational skills. Mentorship should be central, not supplementary, to the process by which purpose can be clarified, competencies internalized, and belonging reinforced. Cross-generational connections between public health professionals ensure that public health leadership is continuously renewed, not episodic, and responsive to shifting contexts. Ten of the 20 articles in this Research Topic 8–11, 13–16, 18 illuminate critical junctures where mentorship animates leadership formation—from undergraduate recruitment to professional training and workforce development.

For example, in academic settings, Johnson et al., in describing the Student Opportunities for AIDS/HIV Research program, outline a cohort-based “mentorship ecosystem” whereby multi-tiered mentors guide students through progressive developmental stages of professional identity formation that ultimately connect them to mission-driven communities. McHale et al. demonstrate how problem-based learning in MPH curricula strengthens leadership capacity for both students and instructors; the process supports not only learners but also educators. Phillips et al. further show that mentored practice environments strengthen student attainment of leadership and communication competencies. Wenzel et al. in their applied MPH leadership course, demonstrate statistically significant gains in students' leadership competencies through experiential learning embedded within supportive instructional environments.

Mentorship beyond academic settings can sustain leadership. Magaña and Benjamin emphasize inclusive leadership strategies to build a resilient public health future. McDonnell et al. highlight investments in the next generation of maternal and child health leaders. Gines et al. note that Mentorship supports early career confidence, while leadership builds later career meaning to reinforce calling across professional stages for Community Health Workers. Internationally, Reng et al. show that culturally-centered mentorship models can measurably strengthen leadership capacity.

## Community and equity

Leadership must embrace communities through authentic partnership, shared power, and sustained commitment to equity. Carman and Pendergrass note that community health improvement efforts such as MAPP 2.0, should help support leaders who are committed to relational trust-building; the authors revisit the Public Health 3.0 vision of the Chief Health Strategist as a convener who builds legitimacy across sectors and fosters collaborative governance. Clayton et al. further emphasize that only community ownership and shared decision-making, not centralized authority, can advance health equity.

Several contributions extend this focus. Chu and Marrero demonstrate that public health leadership can shift from expert-driven intervention to humility and co-creation when leaders focus on a sustained “posture of a learner” with proximity to communities. Hernandez and Murcia advocate for culturally responsive and interdisciplinary approaches that honor lived experience as a legitimate source of knowledge. Gines et al. identify structural and generational barriers facing community health workers, highlighting the importance of mentorship, advocacy support, and inclusive leadership environments. Magaña and Benjamin argue that resilient public health systems depend on leaders embedded in, and trusted by, the communities they serve. Phillips et al. reinforce that leadership credibility is strengthened through effective communication, health literacy, and sustained public engagement.

When communities are genuine partners, competencies can be validated through cultural humility. Equity is the relational test of public health leadership—measured by trust, shared power, and collective capacity for action. Leaders can ultimately shift the paradigm from “no hope” to “new hope” on behalf of a renewed community (7).

## Conclusion

This Research Topic offers both a conceptual framework and a practical roadmap for public health leadership as a systemic, developmental, and relational enterprise. By integrating inner awareness, structured competencies, developmental pipelines, and equity-centered engagement, it invites educators, practitioners, and policymakers to reflect, debate and prepare for action. We consider leadership development as a core and enduring public health function.

Catalyzing public health leadership requires investment at every level—individual, organizational, educational, and societal. Individuals can be encouraged to prepare to answer the call to serve. Classrooms can cultivate reflective practitioners who can be grounded in hope and mission during uncertainty. Agencies can embed mentoring into workforce development. Systems can center community voice as essential, not optional. In the midst of the challenges of our times, we hope this Research Topic can inspire public health leaders everywhere to repair, rebuild and revitalize the field. Doing so can help emerging leaders reimagine what is possible for the future of public health.

## References

[1] Koh HK Jacobson M. Fostering public health leadership. J Public Health (Oxf). (2009) 31:199–201. doi: 10.1093/pubmed/fdp032 19451343

[2] Kouzes JM Posner BZ. Encouraging the Heart: A Leader's Guide to Rewarding and Recognizing Others. San Francisco, CA: Jossey-Bass (1999).

[3] Rev CMD. William Sloane Coffin Dies at 81; Fought for Civil Rights and Against a War. The New York Times (2006). Available online at: https://www.nytimes.com/2006/04/13/us/rev-william-sloane-coffin-dies-at-81-fought-for-civil-rights-and-against.html (Accessed March 10, 2026).

[4] Gupta AK. Bridges across humanity: different religions, similar teachings. New Delhi: Rupa Publications India Pvt. Ltd. (2023).

[5] Koh HK Tso CC Dougherty CP Lazowy EE Heberlein CP Phelps FA. Exploring the spiritual foundations of public health leadership. Front Public Health. (2023) 11:1210160–13. doi: 10.3389/fpubh.2023.1210160 37954055 PMC10634334

[6] Koh HK. Educating future public health leaders. Am J Public Health. (2015) 105:S11–3. doi: 10.2105/AJPH.2014.302385 25706004 PMC4339986

[7] Koh H. Leadership in public health. J Cancer Educ. (2009) 24:11–8. doi: 10.1080/08858190903400385 PMC7097219 20024817

