# Supplementary material for: Editorial: Catalyzing public health leadership research, practice, education, and training
Source: Front Public Health. 2026 May 4;14:1851887. doi: 10.3389/fpubh.2026.1851887 (PMC13180873; doi:10.3389/fpubh.2026.1851887)
Supplement: Supplementary file 1 [file Table_1.docx]

**APPENDIX 1: Public Health Leadership Articles by Category**

**Meaning, Values, Purpose: Spiritual Leadership**

1. Chyu, L., Grinshteyn, E. G., Vian, T., & Godfrey, T. S. J. (2025). *Public health leadership: A framework inspired by timeless lessons from 500 years of the Jesuit tradition*. *Frontiers in Public Health, 13*, 1621381. <https://doi.org/10.3389/fpubh.2025.1621381>
2. Fry, L., Wei, B., Phelps, F., Siegrist, R., Bean, W., Witherell, T. J., & Koh, H. K. (2025). *The relevance of spiritual leadership to public health: Values, meaning and purpose*. *Frontiers in Public Health*.<https://doi.org/10.3389/fpubh.2025.1632959>

**Data-Driven Foundation**

1. Dubois, C., Dissen, A., Bowen, M., Kurz, R., Foster, A., & Buys, D. (2025). *Leadership tasks in public health: Findings from the National Board of Public Health Examiners’ job task analysis*. *Frontiers in Public Health, 13*, 1583383.<https://doi.org/10.3389/fpubh.2025.1583383>
2. Gines, V., Rodriguez, J., Lobaina, D., Sacca, L., Torres, M., & Mejia, M. C. (2025). *Generational perspectives and advocacy barriers among community health workers: Implications for public health workforce leadership*. *Frontiers in Public Health, 13*, 1616506. https://doi.org/10.3389/fpubh.2025.1616506
3. Hamer, D., Gustafson, M., Ortiz Gumina, C., Landis, D. C., Bockelman, P., Perry, E., & Matthews, S. D. (2025). *Bridging the gap: Enhancing data science and leadership knowledge and skills in the context of the public health workforce*. *Frontiers in Public Health, 13*, 1505869.<https://doi.org/10.3389/fpubh.2025.1505869>
4. Kirkland, C., Westfall, N. Y., Patel, K., McCall, T. C., & Leider, J. P. (2025). *New local health officials: Health departments’ newest leaders*. *Frontiers in Public Health, 13*, 1597909.<https://doi.org/10.3389/fpubh.2025.1597909>
5. Popalis, M., Leider, J. P., Mason, A., Najjar, M., Robins, M., & Castrucci, B. (2025). *Bridging the leadership gap: Agency training and support shapes non‑supervisors’ perceptions of their leaders*. *Frontiers in Public Health, 13*, 1610400.<https://doi.org/10.3389/fpubh.2025.1610400>
6. Reng R, Mamven M, Kyari F, Christian E, Orji A, Abah R, et al. Advancing Public Health Leadership through Culturally Centered and Responsive Research Mentorship Training in Nigeria. *Front Public Health*. (2025). <https://doi.org/10.3389/fpubh.2025.1611853>
7. Zweigenthal, V., Christofides, N., Dlungwane, T., Matlala, S. F., Mokgatle, M. M., Opare, A., Patrick, S. M., Schaay, N., Shung‑King, M., Tshitangano, T., & Rispel, L. (2025). *Perceptions of South Africa’s Master of Public Health graduates on the degree’s contribution to their leadership at work and in society*. *Frontiers in Public Health, 13*, Article 1620477. https://doi.org/10.3389/fpubh.2025.1620477

**4P Framework & Key Competencies**

1. Burke, E. M., Fox, J. A., Tager, K., McDowell, S., Phelps, F., & Koh, H. (2025). *Toward a public health leadership national training agenda: A review of conceptual frameworks and core competencies.* Frontiers in Public Health, 13, 1630046.<https://doi.org/10.3389/fpubh.2025.1630046>

**Mentorship & Education**

1. Gines, V., Rodriguez, J., Lobaina, D., Sacca, L., Torres, M., & Mejia, M. C. (2025). *Generational perspectives and advocacy barriers among community health workers: Implications for public health workforce leadership*. *Frontiers in Public Health, 13*, 1616506. https://doi.org/10.3389/fpubh.2025.1616506
2. Hamer, D., Gustafson, M., Ortiz Gumina, C., Landis, D. C., Bockelman, P., Perry, E., & Matthews, S. D. (2025). *Bridging the gap: Enhancing data science and leadership knowledge and skills in the context of the public health workforce*. *Frontiers in Public Health, 13*, 1505869.<https://doi.org/10.3389/fpubh.2025.1505869>
3. Hernandez, M. S., & Murcia, R. (2025). *Culturally responsive approaches to cultivate care and innovation among emerging public health leaders for ethical community engagement: Perspectives informed through lived experience*. *Frontiers in Public Health, 13*, Article 1602187. https://doi.org/10.3389/fpubh.2025.1602187
4. Johnson, G. L., Neubauer, L. C., Bennett, H., Bolivar, A., Kirkland, A. R., & Harper, G. W. (2025). *The student opportunities for AIDS/HIV research program: Promoting public health leadership and transformation for undergraduate students through a principles‑driven, cohort‑based model*. *Frontiers in Public Health, 13*, Article 1601175. <https://doi.org/10.3389/fpubh.2025.1601175>
5. Magaña, L., & Benjamin, G. C. (2025). *Now more than ever: Building a resilient public health future through inclusive leadership*. *Frontiers in Public Health, 13*, 1642510.<https://doi.org/10.3389/fpubh.2025.1642510>
6. McDonnell, K. A., Percy, J., Anders, L., Brown, M. J., Richman, A. R., Deardorff, J., Ruiz, M. S., Liu, J., Russell, K., Snyder, A., & Marshall, C. (2025). *Investing in the development of the next generation of MCH leaders*. *Frontiers in Public Health, 13*, 1606108.<https://doi.org/10.3389/fpubh.2025.1606108>
7. McHale, A., Excellent, M. L., Fleming, W. O., & Upshaw, V. M. (2025). *Shaping future leaders: Developing an MPH leadership curriculum through problem‑based learning*. *Frontiers in Public Health, 13*, Article 1612610.<https://doi.org/10.3389/fpubh.2025.1612610>
8. Phillips, S., Hamilton, J., McCullough, W., El Reda, D., Currier, C., & Clements, J. (2025). *Refocusing public health training on effective leadership and communication skills to increase efficacy*. *Frontiers in Public Health, 13*, 1601444. <https://doi.org/10.3389/fpubh.2025.1601444>
9. Reng R, Mamven M, Kyari F, Christian E, Orji A, Abah R, et al. Advancing Public Health Leadership through Culturally Centered and Responsive Research Mentorship Training in Nigeria. *Front Public Health*. (2025). <https://doi.org/10.3389/fpubh.2025.1611853>
10. Wenzel, S. G., Ky, P., Holt, N., & Austin, E. L. (2025). *Building leadership skills through applied learning in an MPH program*. *Frontiers in Public Health, 13*, 1610306.<https://doi.org/10.3389/fpubh.2025.1610306>

**Community & Equity**

1. Carman, A. L., & Pendergrass, M. E. (2025). *The role of the Chief Health Strategist in community health improvement: A MAPP 2.0 counterproposal.* Frontiers in Public Health, 13, 1601406.<https://doi.org/10.3389/fpubh.2025.1601406>
2. Chu, J. C., & Marrero, A. (2025). *Posture, proximity, and positionality: The power of community engaged service‑learning in public health leadership education*. *Frontiers in Public Health, 13*, 1605757. <https://doi.org/10.3389/fpubh.2025.1605757>
3. Clayton, A., Lawrence, B., & Gousse, T. (2025). *Commentary: The role of the Chief Health Strategist in community health improvement: A MAPP 2.0 counterproposal*. *Frontiers in Public Health*.<https://doi.org/10.3389/fpubh.2025.1711850>
4. Hernandez, M. S., & Murcia, R. (2025). *Culturally responsive approaches to cultivate care and innovation among emerging public health leaders for ethical community engagement: Perspectives informed through lived experience*. *Frontiers in Public Health, 13*, Article 1602187. https://doi.org/10.3389/fpubh.2025.1602187
5. Gines, V., Rodriguez, J., Lobaina, D., Sacca, L., Torres, M., & Mejia, M. C. (2025). *Generational perspectives and advocacy barriers among community health workers: Implications for public health workforce leadership*. *Frontiers in Public Health, 13*, 1616506. https://doi.org/10.3389/fpubh.2025.1616506
6. Magaña, L., & Benjamin, G. C. (2025). *Now more than ever: Building a resilient public health future through inclusive leadership*. *Frontiers in Public Health, 13*, 1642510.<https://doi.org/10.3389/fpubh.2025.1642510>
7. Phillips, S., Hamilton, J., McCullough, W., El Reda, D., Currier, C., & Clements, J. (2025). *Refocusing public health training on effective leadership and communication skills to increase efficacy*. *Frontiers in Public Health, 13*, 1601444. <https://doi.org/10.3389/fpubh.2025.1601444>
